# Cytotoxicity of purified listeriolysin O on mouse and human leukocytes and leukaemia cells

**DOI:** 10.1186/1472-6750-14-77

**Published:** 2014-08-18

**Authors:** Radosław Stachowiak, Marcin Łyżniak, Maja Grabowska, Katarzyna Roeske, Tomasz Jagielski, Jacek Bielecki, Bożena K Budziszewska, Grażyna Hoser, Jerzy Kawiak

**Affiliations:** 1 Department of Applied Microbiology, Institute of Microbiology, Faculty of Biology, University of Warsaw, I. Miecznikowa 1, 02-096 Warsaw, Poland; 2 Medical Centre of Postgraduate Education, Marymoncka 99/103, 01-813 Warsaw, Poland; 3 Department of Hematology, Institute of Hematology and Transfusion Medicine, I. Gandhi 14, 02-776 Warsaw, Poland

**Keywords:** Listeriolysin O, Protein purification, Cytotoxicity, Leukocytes, Leukaemia

## Abstract

**Background:**

Listeriolysin O (LLO) is the main virulence factor of *Listeria monocytogenes* and facilitates the intracellular survival of the pathogen. Some of its characteristics endorse the growing popularity of LLO for use in biotechnology, particularly in the development of novel vaccines. Here, we evaluate the use of LLO to eradicate leukaemia cells.

**Results:**

A purified LLO preparation was obtained by affinity chromatography. The LLO preparation procedure was optimized and purified LLO was tested for optimal conditions of storage including temperature, application of proteinase inhibitors and serum components. We demonstrated the possibility of regulating LLO activity by adjusting cell membrane cholesterol content. The LLO preparation had haemolytic activity and had a cytotoxic effect on the human T-leukaemia Jurkat cell line as well as mouse and human peripheral blood mononuclear cells.

**Conclusions:**

LLO has a very potent cytotoxic activity towards human leukocytes. Importantly, the cytotoxic activity was easily regulated *in vitro* and could be restricted to areas containing malignant cells, raising the possibility of future clinical application of LLO for leukaemia treatment.

## Background

Most bacterial pathogens produce toxins, which are important virulence factors. Some toxins act on cytoplasmic membranes, whereas others are receptor-targeted toxins or membrane damaging toxins. The latter toxins are referred to as cytolysins, and are produced by numerous Gram-positive and Gram-negative bacteria. Some of these toxins display enzymatic activity, while others have cytolytic capacity without enzymatic activity. Occasionally those two mechanisms may act together. For example some phospholipases facilitate the action of pore-forming cytolysins by hydrolysing membrane lipids [1].

Pore-forming cytolysins are a class of membrane-damaging toxins without enzymatic activity. They act by the insertion of their hydrophobic regions into the cell membrane phospholipid bilayer, effectively disrupting the target cells. The most homogenous and numerous group of membrane pore-forming cytolysins are Cholesterol Dependent Cytolysins (CDC). CDCs are produced mostly by Gram-positive bacteria and share similar amino acid sequences and biochemical properties [2]. *Listeria monocytogenes* is an intracellular bacterial pathogen whose important virulence determinants are secreted toxins, one of which is listeriolysin O (LLO), a cytolysin encoded by the *hly* gene belonging to the CDC family. This protein is crucial for pathogen survival within the cytoplasm of the infected cell [3,4].

There have been several attempts to develop effective LLO purification methods using recombinant *Escherichia coli* strains [5-9]. Previously, we attempted to use modified bacteria species enclosed within capillary membranes using *in vitro* experiments with human T leukaemia Jurkat cells [10]. Here we present an improved LLO purification protocol and the results of *in vitro* experiments to determine the haemolytic and cytotoxic activity of purified LLO on peripheral blood leukocytes. The concentration-dependent activity of purified LLO was tested on a human T cell leukaemia cell line (Jurkat) and on normal peripheral blood mononuclear cells (PBMC). This study might provide useful information for future *in vivo* testing of LLO.

## Results

### Purification of Listeriolysin O

**Figure 1:**
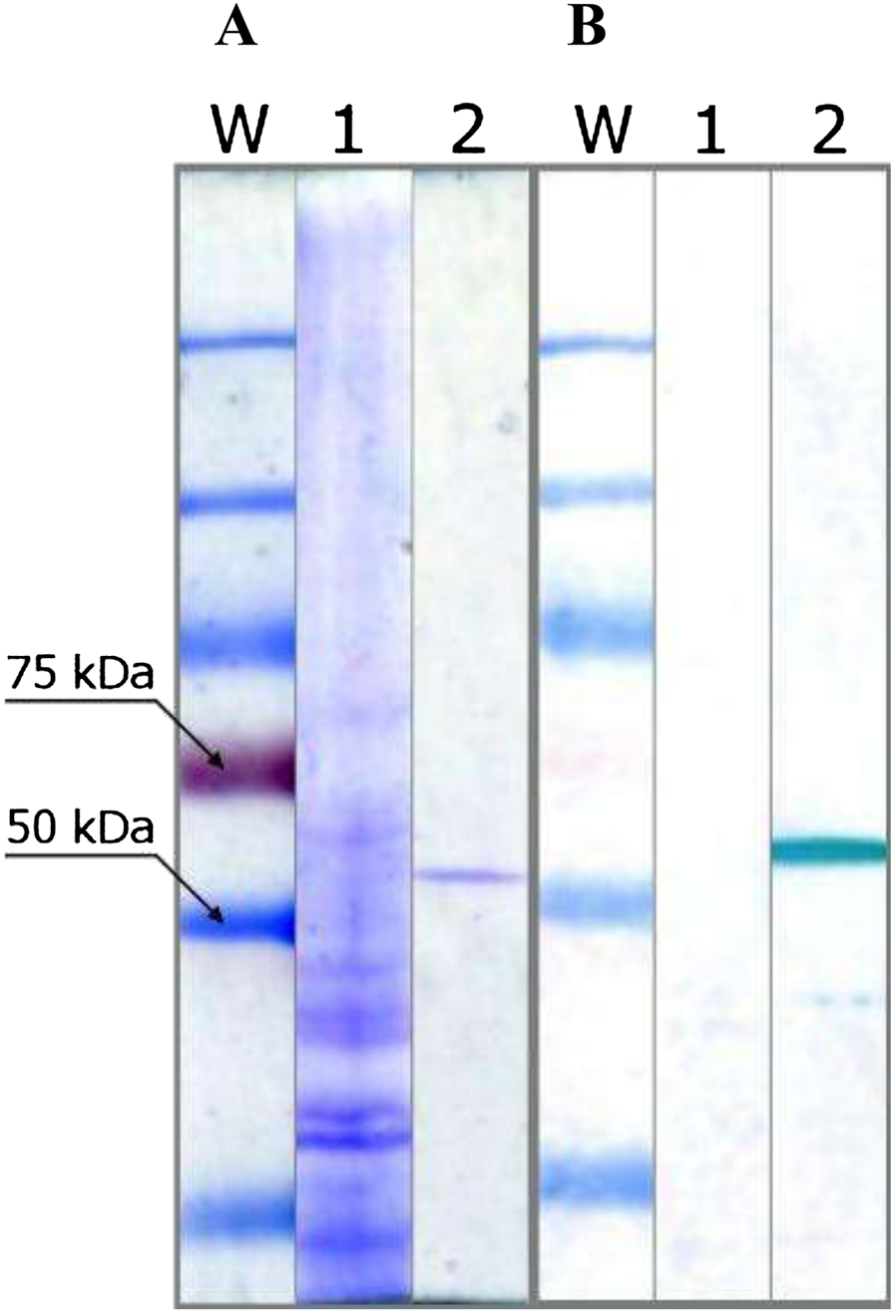
**Electrophoresis analysis of LLO samples.** Total protein (1), electrophoresis **(A)**, and western blot **(B)**. The purified LLO preparation (2) shows one band similar to immunochemical reaction with anti-LLO antibodies.

**Figure 2:**
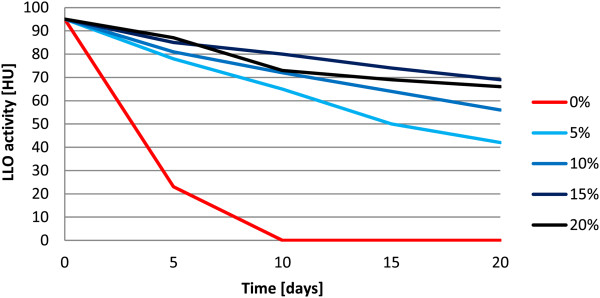
**Effect of glycerol concentrations on LLO stability.** LLO samples were supplemented with varying GOL concentrations (0–20%) and frozen. The haemolytic activity was checked regularly at 5 days intervals.

**Figure 3:**
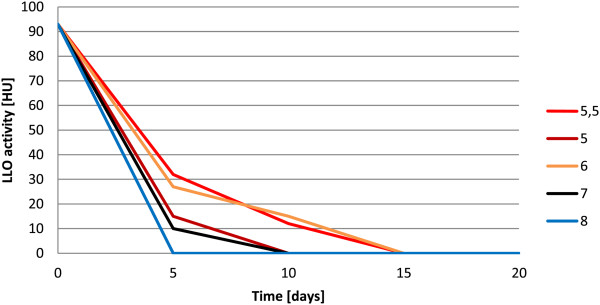
**Effect of pH on LLO stability.** LLO samples were set to different pH values (from 5 to 8) and frozen. The haemolytic activity was checked regularly at 5 days intervals.

The synthesis and affinity purification of His-tagged LLO from *E. coli* harbouring a pET29b-hly plasmid was optimized with a set of buffers with the following gradient: buffer pH ranging from 5 to 8 and NaCl concentration from 0 to 0.5 M. The concentration of imidazole ranged from 0 to 100 mM (for the column buffer) and from 0.25 to 1 M (for the elution buffer). Final protocol and optimal buffer composition are described in the Methods. Notably, lowering the pH of the elution buffer from 8 to 6 allowed a four-fold reduction of the imidazole concentration without a detectable loss of efficiency as compared to the original elution buffer (1 M imidazole, pH 8). The analysis of electrophoretically separated *E. coli* lysates and purified LLO preparation was performed. The results of SDS-PAGE for LLO purified fractions and western blotting results with anti-LLO antibodies are presented in Figure 1. Total protein electrophoresis (Figure 1A) of *E. coli* sonicate (lane 1), purified LLO preparation (lane 2), and western blotting of purified LLO preparation with anti-LLO antibodies (right B 1 and 2 lanes) suggested the presence of a highly uniform protein preparation.The presence of a single protein of approximately 58 kDa, equivalent to the LLO molecular mass was observed. The cytotoxic potential of the purified LLO preparation, prior to application on a Jurkat cell line, was tested on SRBC (sheep red blood cells) for haemolytic activity assay. For most experiments preparations were standardized as follows: the LLO concentration was set at 1.5 μg/ml and samples (3000 HU/ml) were stored at −70°C. To facilitate its application, conditions for longer storage were optimized. The following compounds at various concentrations were tested: glycerol (from 0 to 20%), buffer pH (from 5 to 8), cysteine (from 0 to 10 mM), EDTA (0 to 20 mM) and AEBSF - fluoro 4-(2-aminoethylo)-benzenesulphonyl.HCl (0 to 2 mM). The results for the effects of glycerol and pH on LLO stability are presented in Figures 2 and 3, respectively.

The most potent concentration of glycerol (15%) and pH value (6) were used for all further experiments. For the remaining reagents tested, the differences in their ability to preserve LLO activity were less distinguishable (data not shown). However, the presence of protease inhibitors was necessary to preserve optimum LLO activity. We concluded that the following conditions for LLO preparation storage were optimal for the purified LLO preparation: 1 mM AEBSF, 10 mM EDTA, 15% glycerol, 5 mM cysteine-HCl, pH 6 and a temperature of −70°C. We observed that even after several months of storage, LLO samples retained significant activity.

### Cytotoxic activity

**Figure 4:**
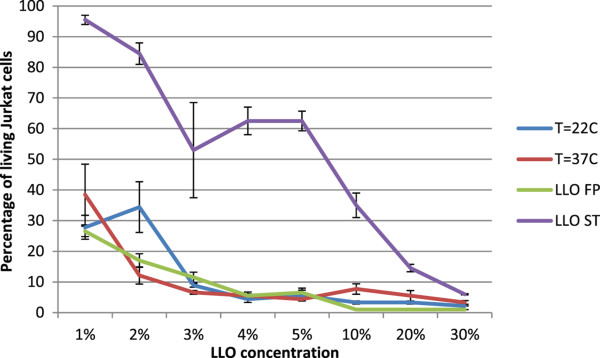
**Activity of purified LLO on Jurkat cell line.** LLO concentration dependence, temperature dependence and conditions of storage were compared. Presented values show the percentage of living cells after exposure to LLO. Cytotoxicity of LLO at 22°C and 37°C were compared on preparations without protease inhibitors, and the influence of storage for 25 days (at −70°C) of LLO preparations on cytotoxicity in the presence of protease inhibitors was assessed. FP –freshly prepared LLO preparation; ST- stored LLO preparation. Median values; P_25_ and P_75_, n = 3, p < 0.0001 between FP and ST. Differences in cytotoxic activity at 22°C and 37°C were not significant.

**Figure 5:**
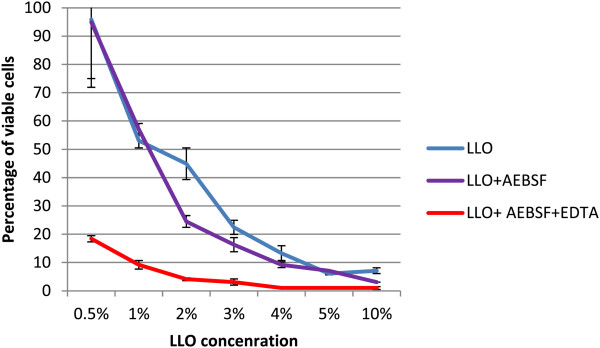
**LLO stability in the presence of protease inhibitors tested on Jurkat cells incubated in RPMI 1640 medium containing 4% inactivated NCS.** Presented values show percentage of living cells after exposure to LLO. Median value, P_25_ and P_75_, n = 3, p < 0.0001 compared with controls without inhibitors.

The cytotoxicity of the purified LLO preparation was tested on a Jurkat cell line at 22°C and 37°C (Figure 4). The cytotoxic activity of LLO was concentration-dependent and was similar at both temperatures under the test conditions employed. Storage of the LLO preparation even in the presence of protease inhibitors and 10% glycerol at −70°C significantly decreased the preparation activity (p < 0.0001) (Figure 5). However, even the addition of 3% glycerol stored purified LLO preparation killed 50% of Jurkat cells and the presence of 30% glycerol purified LLO preparation killed 95% of the cells.

### LLO activity in culture medium containing cholesterol

**Figure 6:**
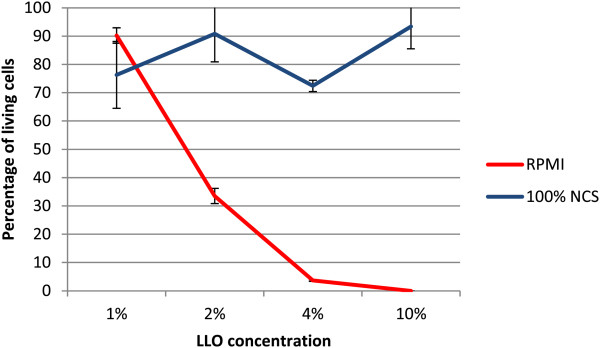
**Effect of serum containing cholesterol on LLO cytotoxicity.** Cytotoxicity assay on a Jurkat cell line in RPMI-1640 without NCS and in 100% inactivated NCS with PI. NCS contained 390 μM (150 mg/dl) of cholesterol. Results show percentage of living cells. LLO concentration dependence was compared. Median values; P_25_ and P_75_, n = 4, p < 0.0001.

**Figure 7:**
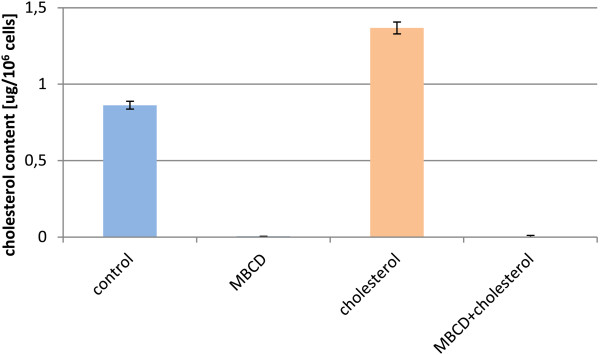
**Cholesterol content in Jurkat cells after cholesterol and MβCD treatment.** Jurkat cells were incubated for 1 h at 37°C in RPMI1640 containing 26 μM cholesterol or 5 mM MβCD or both these compounds. Results show cholesterol content in μg per 10^6^ cells.

**Figure 8:**
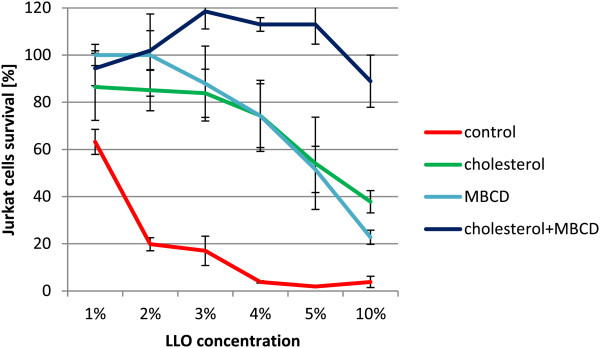
**Effect of cholesterol and MβCD pre-treatment on Jurkat cells survival.** Jurkat cells were preincubated in RPMI 1640 for 1 h at 37°C with cholesterol (26 μM; 10 μg/ml) or MβCD (5 mM) or a mixture of both. Then LLO was added and the sample was incubated for 30 min. LLO cytotoxicity was tested on a Jurkat cell line with PI. Values represent percentage of living cells. LLO concentration dependence was compared. Median values; P_25_ and P_75_, n = 4 p < 0.004 between control and cholesterol or MβCD groups.

The cholesterol dependency of LLO was tested. The cytotoxicity of the LLO preparation was tested on Jurkat cell line in NCS (neonatal calf serum) medium containing cholesterol (390 μM) (Figure 6). The results showed significant inhibition (p < 0.0001) of the killing properties of purified LLO in medium composed of serum containing cholesterol.The dependence of LLO activity on cholesterol is well known. Cholesterol in cell membranes is assumed to act as a receptor for LLO, and an insufficient amount of cholesterol content within cell membranes may be inhibitory. Similarly, the presence of cholesterol in incubation medium may bind to LLO, which in turn may prevent its binding to cell membranes. The cell membrane cholesterol content may be diminished by the addition of methyl-β-cyclodextrin (MβCD) to culture medium. This was tested by preincubation of Jurkat cells for 1 h at 37°C in RPMI1640 containing 26 μM cholesterol or 5 mM MβCD or both of these compounds. The effect of these reagents on membrane cholesterol content was verified by cholesterol assay. As anticipated, the addition of cholesterol increased its membrane content while incubation with MβCD completely removed cholesterol from cellular membranes (Figure 7).Jurkat cells preincubated with modified cholesterol content were subsequently incubated with purified LLO for 30 min the survival rate of the cells was assessed (Figure 8).

The cholesterol concentration dependence on LLO was compared with RPMI1640, and RPMI1640 containing 26 μM cholesterol or 5 mM of MβCD. Statistically significant (p < 0.004) differences were noted between cholesterol or MβCD samples and the controls. Interestingly, in medium containing 5% LLO and cholesterol, up to 54% of Jurkat cells survived. A similar percentage of cell survival (51.5%) was observed in the presence of MβCD in the medium as compared with control medium where only 2% of Jurkat cells survived. When both factors were present almost all cells survived, and the cytotoxic activity of LLO was completely blocked. Here the MβCD molar concentration was about 200 times higher than the cholesterol concentration.

### Sensitivity of human and mouse PBMC to the purified LLO preparation

**Figure 9:**
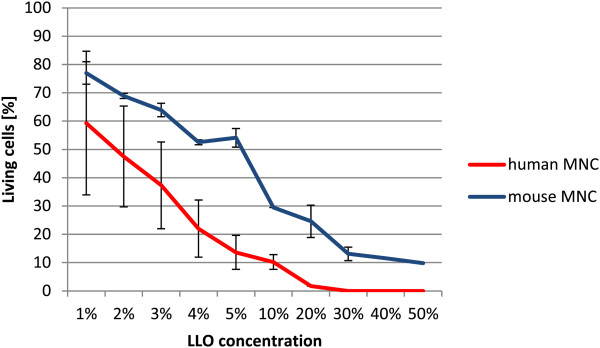
**Comparison of human and mouse PBMC sensitivity to LLO cytotoxicity.** Results of purified LLO cytotoxicity tested on human and mouse PBMC (1 × 10^6^/ml) in RPMI 1640 containing 4% NCS with PI. The percentage of living cells is shown. LLO concentration dependence was compared. Median values; P_25_ and P_75_, n = 3, p < 0.005.

Human or mouse PBMC were isolated, diluted to a concentration of 1 × 10^6^ cells/ml, incubated in RPMI 1640-10% NCS with protease inhibitors and their survival rate tested after incubation with the purified LLO preparation. Concentration dependence survival was observed (Figure 9) in a cytometric test with propidium iodide (PI). Statistically significant differences for human and mouse PBMC sensitivity to LLO was observed (p < 0.005). Human cells were more sensitive than mouse cells: at 5% purified LLO concentration 54% of mouse PBMC survived, in contrast to only 14% of human cells.

## Discussion

In this study, an *E. coli* strain expressing his-tagged LLO was used, similar to previous reports. However, in contrast to those studies, which used standard elution buffer (pH 8) with one exception [7], here we proposed a modified procedure for LLO purification. To the best of our knowledge, this is the first application of an acidic buffer for the purification of LLO with nickel - nitrilotriacetic acid (Ni-NTA) resin. The use of low pH buffer offers two advantages over commonly used pH 8 elution buffer: (i) it effectively reduces the ability of Ni-NTA resin to bind proteins, facilitating the elution of a higher protein yield with lower imidazole concentrations, and (ii) it preserves the activity of the LLO protein. Given that all CDCs share similar biochemical features and have a similar overall structure [2], the purification method described here should be applicable for other CDCs. Storage conditions are, however, most likely restricted to Listerial cytolysin due to its unique characteristics [3,4]. The storage conditions that promoted the stability of LLO haemolytic activity and cytotoxic activity were confirmed using sheep erythrocytes and Jurkat cells, respectively. However, the positive effect of protease inhibitors on the stability of the purified LLO preparation suggested the presence of active proteases in the purified preparation of LLO.

It was shown that purified LLO demonstrates the properties of an authentic *Listeria monocytogenes* protein. Listeriolysin O is crucial for pathogen survival within the cytoplasm of infected cells [3,4]. Less well known is the activity dependence of purified LLO when applied outside the cell, on the cell membrane. The cell membrane might not be as uniform as predicted by the classical fluid mosaic model, and could contain differently arranged regions. The lipid raft hypothesis describes membrane areas stabilized by cholesterol within a liquid-ordered phase. These areas are engaged in the regulation of cell adhesion, transport, sorting of lipids and proteins and signal transduction. Membrane lipid rafts are regions where cholesterol and marker proteins such as CD59 are concentrated [11,12]. While cholesterol acts as a receptor for all CDCs, CD59 is considered an additional receptor for two CDC toxins [13]. Possibly, other CDCs also require additional factors, which might explain some long-observed differences in different cells susceptibility to CDCs activity. Human leukocytes were significantly more sensitive than mouse cells to LLO activity. This might be simply a result of the different overall sensitivity of human and mouse cells or a specific difference between cellular receptors, possibly lipid rafts. Very subtle differences at the molecular level might be of great importance, i.e. the difference of a single amino acid for Internalin (Inl) A–E-cadherin interactions. Human cadherin is preferentially targeted by the InlA surface protein [14]. *L. monocytogenes* is better adopted to infect humans than mice and might explain its high activity towards leukocytes that normally threaten *L. monocytogenes* survival in human cells.

The cytotoxic activity of purified LLO was observed when applied to human leukocytes in the presence of a known concentration of cholesterol and/or MβCD in the medium, which removed cholesterol from cell membranes. We concluded that the presence of cholesterol in cell membranes and in the culture medium modified the cytotoxic activity of purified LLO. During *in vivo* conditions, the presence of cholesterol is expected in peripheral blood plasma; however, it may also be present within the tissue matrix [15]. The LLO preparation may have application when locally administered directly into tissues, e.g. subcutaneously, intraperitoneally, intraorganally or intratumourally. We demonstrated the possibility of simple regulation of LLO cytotoxic activity *in vitro* by the additional application of cholesterol or MβCD. This might also have practical significance *in vivo* although the safety of such a treatment might be an issue. However, it might be possible since cyclodextrins are already in clinical use [16]. The LLO preparation has haemolytic activity and cannot be applied intravenously; however its intraperitoneal or subcutaneous application is possible. The relatively low activity and stability of LLO in physiological conditions should be advantageous and allow the restriction of cytotoxic activity to selected regions, thus limiting possible side-effects. However, the specificity of cytotoxin-based anti-cancer therapy remains a great challenge. This could be resolved by combining toxic components with antibodies, producing so called immunotoxins. The most popular immunotoxin components of bacterial origin are exotoxin A derived from *Pseudomonas aeruginosa* and diphtheria toxin from *Corynebacterium diphtheriae*[17,18]. Although its mode of action might appear to preclude the combination of CDCs with antibodies, it was recently shown that directing cytotoxicity using antibodies is applicable for LLO [19]. The cytotoxic features of LLO and the possibility of controlling its activity make it a good immunotoxin candidate, although the direct use of LLO for clinical treatment must be preceded by a detailed description of its activity under the form of purified preparation.

## Conclusions

In this study, we purified LLO by affinity chromatography demonstrating the usefulness of acidic buffer to remove LLO from Ni-NTA resin. The satisfactory storage conditions of the purified LLO preparation were also demonstrated to be an important step before planning use of the toxin. This study underscores the role of cryopreservative glycerol and a thiol-reducing agent, cysteine, for LLO stability and activity. The storage additives in the doses used were not toxic to eukaryotic cells, while LLO displayed potent *in vitro* activity towards leukaemia cells. LLO activity was easily regulated *in vitro* and possibly *in vivo*, and this information might facilitate its future clinical application.

## Methods

### *Escherichia coli* strains and growth conditions

The source of purified LLO was *E. coli* strain BL21(DE3) (Novagen, Madison, Wis) with plasmid pET29B (Novagen) carrying cloned *hly* gene, kindly provided by Dr. Higgins [6]. The bacteria were grown in LB broth (Sterbios, Warsaw, Poland), supplemented with 30 μg/ml kanamycin (Merck, Darmstadt, Germany) at 37°C with 120 rpm shaking. Bacteria were cultured until the optical density reached 1.5. To induce LLO-His synthesis, IPTG (Sigma, Taufkirchen, Germany) was added to a final concentration of 1 mM.

### Eukaryotic cell isolation and growth conditions

The target *Eukaryotic* cells were: SRBC - sheep red blood cells (Biomed, Warsaw, Poland), human acute T cell leukaemia cell line (Jurkat, ATCC TIB 152), and PBMC isolated from humans or mice.

Jurkat cells were grown in RPMI 1640 culture medium (Gibco, Gaithersburg, MD, USA) with 10% of inactivated NCS containing 100 U of penicillin and 0.1 mg of streptomycin per 1 ml or in DMEM medium (Sigma) with 10% of inactivated NCS. Serum contained 150 mg/dl cholesterol, and the final cholesterol concentration in the culture medium was 15 μg/ml (39 μM).

PBMC were cultured, after isolation, *in vitro* for 2–7 days before a cytotoxicity experiment. For human peripheral blood experiments *in vitro*, patients gave their personal consent and the procedure was accepted by the Bioethical Commission at the Medical Center of Postgraduate Education, Warsaw (Feb 27, 2008).

Isolation of PBMC was performed by an overlayer of peripheral blood diluted with PBS (1:1), on 5 ml of Histopaque 1077 (Sigma) preparation in a test tube and centrifugation for 30 min at 540 × *g*. The interlayer of PBMCs consisting of lymphocytes and monocytes was separated and diluted with 10 ml of culture medium, centrifuged for 5 min at 200 × *g* and the washed cells suspended in fresh culture medium were counted in a Burker camera. The concentration of the cell suspension was corrected to a final value of 2 × 10^6^/ml before culturing. The cultures were performed in Falcon vessels (dishes) or in 6-well flat bottom plates in RPMI 1640 with 10% NCS and antibiotics, and half of the medium volume was changed twice weekly.

For some experiments cell cultures were supplemented with the following compounds to modify the LLO activity: cholesterol (Sigma), NCS containing 150 μg/ml cholesterol (390 μM), and MβCD (Sigma).

### The affinity chromatography of LLO

Ten ml of *E. coli* suspension (approximately 10^8^/ml) grown in the presence of 1 mM IPTG was centrifuged at 4400 × *g* for 10 min at 4°C, and the supernatant was removed. The pelleted cells were resuspended in 1 ml of sterile column buffer (50 mM Tris, pH 8, 300 mM NaCl, 50 mM imidazole) and were ruptured by sonication in 3 pulses of 30 s each. Then, the bacterial fragments were removed by centrifugation (7000 × *g*, 15 min). The supernatant was collected, diluted with column buffer and applied onto the affinity column (His-Bind Ni Column, Novagen) previously equilibrated with the column buffer. The column was washed with the same buffer, and then LLO was desorbed with the elution buffer (50 mM MES, pH 6, 300 mM NaCl, 250 mM imidazole). The collected fraction was subsequently dialysed to remove imidazole (50 mM MES, pH 6, 300 mM NaCl, 5 mM cysteine HCl). Protease inhibitors: fluoro 4-(2-aminoethylo)-benzenesulphonyl.HCl (AEBSF, Sigma), EDTA (Sigma), and glycerol (Merck) were added to a final concentration of 1 mM, 10 mM, and 15% (v/v), respectively. Protein concentrations were assayed with NanoOrange® Protein Quantitation Kit (Molecular Probes) using an infinite M200 PRO reader (TECAN, Goring on Thames, UK).

### Western blot analysis of LLO preparations

Electrophoretically resolved proteins (SDS-PAGE) were transferred onto a nitrocellulose membrane using the BIO-RAD Trans-Blot system, according to a protocol recommended by the manufacturer. The membrane was blocked using 5% skimmed milk in Tris-buffered saline buffer (TBS), pH 7.6. Primary rabbit polyclonal anti-LLO antibody (Abcam, Cambridge, MA, USA) and secondary goat polyclonal antibody (Abcam) conjugated with alkaline phosphatase were used at 1:1500 and 1:10,000 dilutions, respectively. The membrane was developed using the NBT/BCIP reagent (Merck).

### Assay of haemolytic activity

Haemolytic activity was assayed using SRBC. The erythrocytes were washed three times with PBS and suspended to a final concentration of 20% (v/v) in PBS (pH 7.4). The LLO preparation was diluted by adding 10 μl of its preparation to a final volume of 1 ml of 2% SRBC suspension in PBS. The prepared solution was incubated for 30 min at 37°C and then centrifuged for 3 min at 150 × *g*. The released haemoglobin was measured spectrophotometrically at λ = 410 nm. Haemolytic Units (HU) were calculated after setting 0 HU as the activity of a negative control, and 100 HU for total haemolysis, observed in samples of erythrocytes lysed with 0.01% SDS.

### Cholesterol assay

Jurkat cells were resuspended in fresh RPMI medium without any additives (control), supplemented with 26 μM cholesterol, with 5 mM MβCD or with both these compounds. The cells were incubated for 1 h at 37°C, washed with PBS three times, counted and disrupted by sonication. Membrane cholesterol was extracted with isopropanol and quantified by a cholesterol assay kit (R-Biopharm, Darmstadt, Germany).

### Cytometric assays

The activities of the LLO preparations were tested on Jurkat cells or PBMC. The Jurkat cell suspension in a culture medium (1 × 10^6^/ml) of 20 μl, was mixed with 30 μl of LLO preparation diluted with physiological salt solution at different proportions, and immediately incubated for 30 min at room temperature (20–22°C). The time of the incubation was assumed to be the same as for the haemolytic assays. The final cell concentration was 0.4 × 10^6^/ml. The effect of the LLO preparation was titrated in an assay volume of 50 μl containing cell suspension (20 μl) and 30 μl of physiological salt solution containing the LLO preparation. The LLO preparation content investigated was: 0.5, 1, 2, 3, 4, 5, 10, 20, 30, 40, and 50% of the original LLO concentration (1.5 ng/μl) with activity set at 3 HU/μl. After cell sample incubation, physiological saline containing PI (1 μg/ml) was added before acquisition by a cytometer. PI penetrates the cell membrane of damaged cells and binds to the cell nucleus. In the fluorescence quadrant readings of forward scatter (FSC)/PI fluorescence, the PI unstained events indicate the percentage of living cells. In the FACSCalibur BD cytometer, 5000 events were collected from each sample and analysed by Cell Quest software (Becton Dickinson, Franklin Lakes, NJ, US). Experiments were repeated at least three times.

### Statistical analysis

The flow cytometry results were presented as median values and percentile values P_25_ and P_75_. Statistical analysis of flow cytometric experiments was in the global linear model (GLM) after logit transformation of values. The *post hoc* test of Tukey was used. Results of haemolytic assays were presented as mean values ± standard deviation (SD). Analysis of variance was performed by ANOVA. Statistically significant values of p < 0.05 were assumed. Statistical software SAS 9.2 was used.

## Competing interests

The authors declare no competing interests.

## Authors’ contributions

RS contributed to experimental strategy, analysed data, conducted experiments, wrote and submitted the manuscript. MŁ performed cytometric assays of eukaryotic cells and drafted the manuscript. MG managed all aspects of the protein purification and optimised the method. KR conducted SDS-PAGE and western-blot analysis. TJ contributed to development of the LLO purification method and edited the manuscript. JB supervised the experimental design and execution and guided manuscript preparation. BB isolated PBMC from patients and cultured the cells. GH isolated and cultured mouse PBMC and managed tissue cultures. JK conceptualized the experimental strategy, supervised cytometric assays and significantly contributed to the writing of the manuscript. All authors read and approved the final manuscript.
